# A multicenter end‐to‐end postal audit of skin markerless surface‐guided radiation therapy

**DOI:** 10.1002/acm2.70893

**Published:** 2026-10-29

**Authors:** Masahide Saito, Ryohei Yamauchi, Hironori Kojima, Yoshinobu Shimohigashi, Takeshi Ono, Yoshifumi Oku, Shinobu Kumagai, Naoki Tohyama, Masahiko Kurooka, Eriko Saito, Masataka Sakamoto, Yusuke Ueshima, Takayuki Kanai, Naoki Hayashi, Tatsunori Saito, Tomoki Kitagawa, Makoto Sasaki, Hiroki Katayama, Hidekazu Suzuki, Chie Kurokawa, Hiroshi Onishi

**Affiliations:** ^1^ Department of Therapeutic Radiology University of Yamanashi Yamanashi Japan; ^2^ Department of Radiation Oncology St. Luke's International Hospital Tokyo Japan; ^3^ Department of Radiology Kanazawa University Hospital Ishikawa Japan; ^4^ Department of Radiological Technology Kumamoto University Hospital; ^5^ Faculty of Life Sciences Kumamoto University Kumamoto Japan; ^6^ Department of Radiology Kagoshima University Hospital Kagoshima Japan; ^7^ Department of Radiology Teikyo University Hospital Tokyo Japan; ^8^ Department of Radiological Sciences Komazawa University Tokyo Japan; ^9^ Department of Radiation Therapy Tokyo Medical University Hospital Tokyo Japan; ^10^ Department of Radiation Oncology Chiba Tokushukai Hospital Chiba Japan; ^11^ Department of Radiology Hamamatsu University School of Medicine Shizuoka Japan; ^12^ Regional Creative Education Center Hamamatsu University School of Medicine Shizuoka Japan; ^13^ Department of Radiation Oncology Tokyo Women's Medical University Tokyo Japan; ^14^ Division of Medical Physics, School of Medical Sciences Fujita Health University Aichi Japan; ^15^ Department of Radiology Seirei Hamamatsu General Hospital Shizuoka Japan; ^16^ Department of Radiation Oncology Aichi Cancer Center Aichi Japan; ^17^ Division of clinical radiology service Kyoto University Hospital Kyoto Japan; ^18^ Division of Clinical Radiology, Department of Medical Technology Kagawa University Hospital Kagawa Japan; ^19^ Department of Radiological Technology, Faculty of Medical Technology Teikyo University Tokyo Japan; ^20^ Faculty of Health Science Juntendo University Tokyo Japan

**Keywords:** breast cancer, postal audit, surface‐guided radiation therapy

## Abstract

**Background:**

Surface‐guided radiation therapy (SGRT) enables skin markerless positioning, but its accuracy may depend on the reference surface, hardware configuration, commissioning, and institutional workflow. Two reference surfaces are commonly used: a DICOM reference generated from planning CT images and an SGRT reference acquired in the treatment room. Multicenter end‐to‐end data across commercial systems remain limited.

**Purpose:**

To evaluate static irradiation accuracy and setup time with two reference surfaces in a nationwide multicenter skin markerless SGRT postal audit.

**Methods:**

A prospective end‐to‐end postal audit was conducted from October 2023 to July 2025 at 40 Japanese institutions using five commercial SGRT systems (AlignRT, Catalyst, ExacTrac Dynamic, IDENTIFY, and VOXELAN), eight per system. Each institution received a common phantom package: a breast‐shaped surface phantom, two cubic phantoms containing gold markers, and radiochromic films. Markerless setup and irradiation were performed with both the DICOM and SGRT references; ExacTrac Dynamic institutions performed the DICOM reference setup only. Static irradiation accuracy was the difference between the film‐derived radiation center and the intended center in the DICOM RT Plan; the primary metric was the three‐dimensional translational vector error (3D vector error). Paired comparisons used the Wilcoxon signed‐rank test, and multivariable linear regression evaluated factors associated with both endpoints.

**Results:**

Critical setup errors exceeding 5 mm occurred at four institutions initially (institution‐level error rate, 10.0%; three with the DICOM and one with the SGRT reference); one DICOM reference setup was not remeasured and remained out of tolerance, and was excluded as an outlier, leaving 39 DICOM and 31 SGRT reference setups for analysis. Excluding ExacTrac Dynamic, the DICOM reference showed a significantly larger 3D vector error than the SGRT reference (1.54 ± 0.91 vs. 1.20 ± 0.61 mm, *p* = 0.043). Setup time did not differ between references (86.19 ± 43.57 vs. 81.61 ± 37.09 s, *p* = 0.804). Axis‐specifically, the difference between references was confined to the anterior–posterior direction (mean absolute difference 0.36 mm, *p* = 0.002), whereas a caudal superior–inferior offset was present with both (−0.50 ± 1.29 and −0.37 ± 0.87 mm; both *p* < 0.01) and did not differ between them (*p* = 0.854). The 1‐camera condition was independently associated with a larger 3D vector error than the 3‐camera condition (β = 0.90 mm; 95% CI, 0.17–1.63 mm; *p* = 0.017); no factor was associated with setup time, which showed no correlation with 3D vector error.

**Conclusions:**

Skin markerless SGRT achieved millimeter‐level static irradiation accuracy but carried a non‐negligible risk of critical setup errors. The difference between the two reference workflows was confined to the anterior–posterior direction, whereas a superior–inferior offset common to both indicates a residual uncertainty in the audit chain itself. Reference surface selection and camera configuration influenced accuracy, whereas setup time was not a reliable surrogate. These findings highlight the importance of commissioning, standardized reference surface acquisition, routine QA, and independent third‐party audits.

## 1 INTRODUCTION

In recent years, surface‐guided radiation therapy (SGRT) has attracted considerable attention as it enables both highly accurate patient positioning and improved quality of life by eliminating the need for skin markings.^1^, ^2^ In particular, in the context of breast irradiation, SGRT has been shown to contribute to the improved management of respiratory motion during treatment and enhanced setup reproducibility, leading to accelerated clinical adoption, particularly in Europe and North America.^3^

Regarding the use of SGRT, the importance of appropriate system calibration and quality assurance (QA) has been repeatedly emphasized in the recommendations of the AAPM Task Group 147,^4^ Task Group 302,^5^ and ESTRO‐ACROP guidelines.^6^ However, commercially available SGRT systems differ in their hardware configurations and algorithms, and their performances may vary depending on the installation environment and clinical workflow. Currently, widely used commercial SGRT systems include AlignRT,^7^, ^8^, ^9^ Catalyst,^9^, ^10^, ^11^ IDENTIFY,^12^, ^13^ and ExacTrac Dynamic,^14^ all of which have been increasingly implemented in radiotherapy facilities worldwide. In Japan, SGRT is increasingly being implemented in clinical practice, and a national survey reported that 26.5% of institutions use skin markerless SGRT.^15^ In addition to the systems mentioned above, the domestically developed VOXELAN system has also been adopted in clinical settings.^16^, ^17^

In SGRT, two types of reference surfaces are generally available: one generated from CT images (DICOM reference) and one acquired in the treatment room using the SGRT system (SGRT reference). Because the choice of the reference surface is directly associated with positioning accuracy, a thorough understanding of the characteristics of each reference surface is essential.^5^ Although comparisons of reference surfaces have been reported in single‐institution studies,^18^ no multicenter studies have systematically evaluated end‐to‐end static irradiation accuracy across all SGRT systems based on differences in reference surface images. Furthermore, as SGRT has been adopted across a wide range of institutional settings with varying levels of experience and diverse hardware configurations, it is critical to assess how the choice of reference surface affects accuracy under real‐world clinical conditions across multiple centers.

Despite this expanding clinical use, the standardization of SGRT practice across institutions remains a concern, as differences in hardware configurations, institutional experience, and workflow protocols may introduce variability in positioning accuracy. A systematic assessment of inter‐institutional accuracy is therefore needed to support quality assurance standardization. Therefore, we conducted a prospective multicenter end‐to‐end postal audit to evaluate static irradiation accuracy and setup time with different reference surfaces, using a common phantom across institutions where various commercial SGRT systems are routinely implemented. The findings of this study are expected to contribute to the standardization of SGRT quality assurance, both in Japan and internationally.

## 2 MATERIALS AND METHODS

### 2.1 Study design

The end‐to‐end postal audit period extended from October 2023 to July 2025 and targeted 40 institutions in Japan that operated one of the five SGRT systems (AlignRT, Vision RT, UK; Catalyst, C‐RAD AB, Sweden; ExacTrac Dynamic, Brainlab AG, Germany; IDENTIFY, Varian Medical Systems, USA; VOXELAN, Electronics Research & Development, Japan), with eight systems evaluated for each device type. This sample represented the majority of institutions with operational SGRT systems in Japan at the time of the study, constituting a near‐census survey of the domestic SGRT‐equipped facilities. The participating institutions were recruited through a nationwide survey questionnaire approved by the Institutional Review Board of St. Luke's International Hospital (approval No. 24‐RC041) and informed consent for participation was obtained.

Each participating institution received a postal phantom package and was instructed to complete the specified procedures within three business days. The instruction manual described phantom assembly, CT scanning, treatment planning, both SGRT setup procedures, film handling and data submission. No additional training was provided. After the completion of the survey, a report was sent to each institution. If a discrepancy of ≥ 5 mm in any direction was identified, the institution was encouraged to perform an optional repeat measurement. For institutions that conducted repeated measurements, only the post‐remeasurement results were included in the analyses presented in this manuscript. The 5 mm criterion was the action level of the audit, not a performance tolerance, and is looser than the component‐level tolerances of AAPM TG‐302 (2 mm, and 1 mm for SRS/SBRT) and the ESTRO‐ACROP guideline (2 mm and 1°); performance against those is reported in the Results.

### 2.2 Postal audit phantom and study workflow

The detailed structure of the phantom is illustrated in Figure 1(a). Each participating institution received a package containing a breast‐shaped surface phantom, two cubic phantoms (Cube A for DICOM reference setup and Cube B for SGRT reference setup), a written instruction manual, a data storage disk, and a prepaid return shipping label. Both cubic phantoms were identical in external dimensions (15 × 15 × 15 cm^3^). Both came from one drawing in a single lot and are nominally identical in dimensions, marker coordinates and film‐slot geometry (tolerance ± 0.2 mm). Two cubes were used because a film set can be exposed only once. Each cube contained embedded gold markers for image‐guided alignment and radiochromic films inserted on four surfaces, allowing the calculation of the irradiation isocenter coordinates through image analysis. Each beam crossed two parallel films separated along its axis, so the four field centers over‐determine the radiation center and all rotations. The breast‐shaped surface phantom was mounted on the upper face of each cube to provide a breast‐like surface for SGRT recognition. Its underside had a 10 mm recess that seated on the cube without play in one orientation; it was detached once per institution. The isocenter lay 15 mm cranial to the cube center and 75 mm posterior to its anterior face, and the surface apex about 64 mm anterior to that face, giving roughly 139 mm from the registered surface to the isocenter — a deep‐seated target, not a clinical breast isocenter. Details of the phantom design and image analysis methodology have been described previously.^18^

**FIGURE 1:**
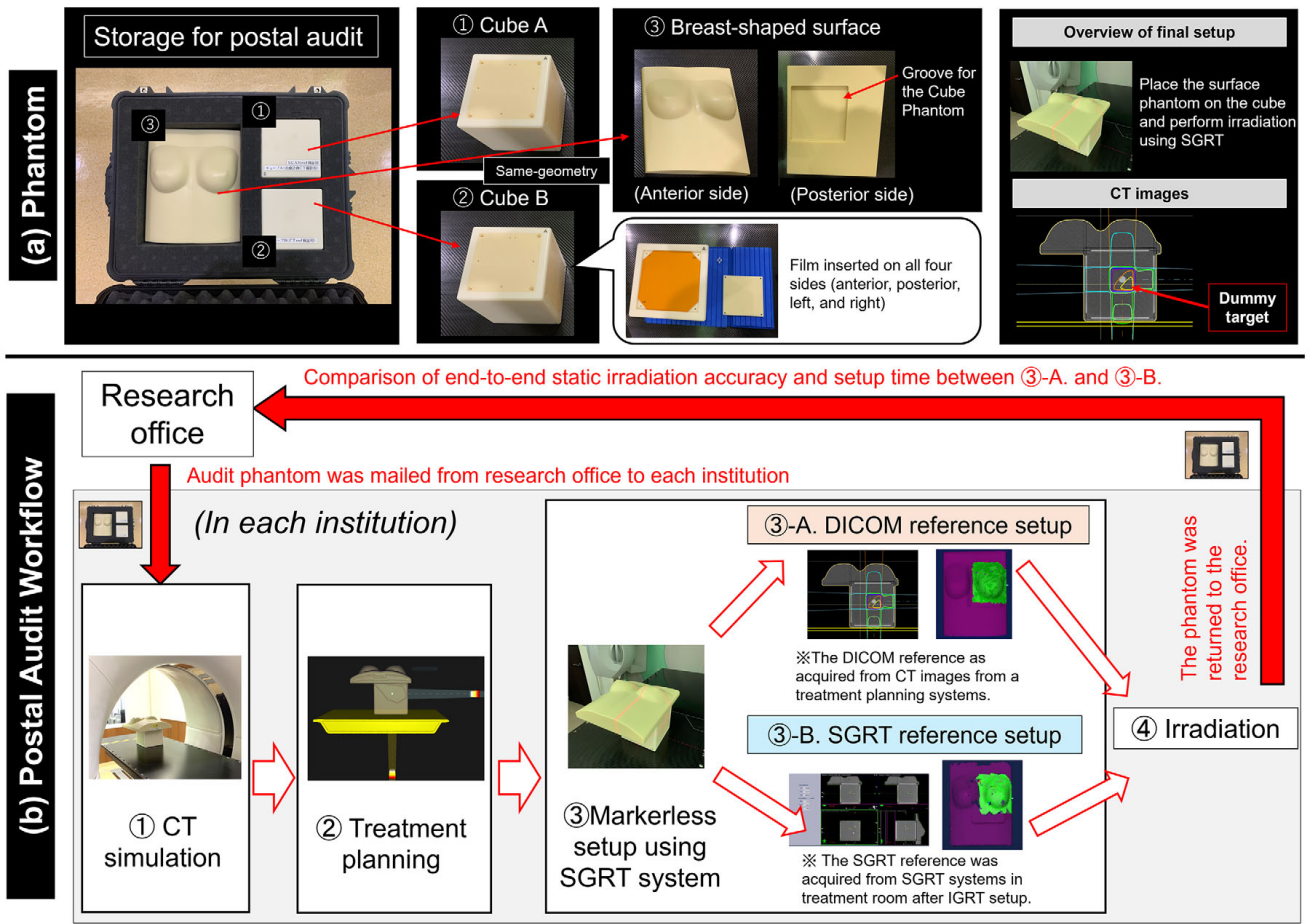
Postal audit phantom and workflow. (a) The phantom package: ① Cube A and ② Cube B, two cubes of identical geometry (15 × 15 × 15 cm^3^) with embedded gold markers and radiochromic films inserted on four faces (anterior, posterior, left and right), and ③ a breast‐shaped surface phantom whose groove seats on the cube; the right‐hand panels show the assembled phantom and the planning CT with the dummy target. (b) Procedure at each institution: ① CT simulation, ② treatment planning, ③ markerless setup with the SGRT system using either ③‐A the DICOM reference or ③‐B the SGRT reference, and ④ irradiation. The phantom was mailed from, and returned to, the research office, which compared static irradiation accuracy and setup time between ③‐A and ③‐B.

Radiochromic films (RTQA2) were digitized with a flat‐bed scanner (ES‐G11000, Epson, Tokyo, Japan) at 300 dpi, corresponding to a pixel size of 0.085 mm. Repeated scanning of the same film set (*n* = 3) varied by less than the 0.01 mm and 0.01° reporting precision; repetition of the whole procedure (*n* = 3) gave standard deviations of 0.15 mm and 0.05°, and the gold‐marker and film‐derived centers differed by 0.4 ± 0.2 mm.

The evaluation endpoints of this study were (1) static irradiation accuracy, defined as the difference between the actual radiation center determined by film analysis and the intended radiation center specified in the DICOM RT Plan, and (2) setup time. Both endpoints were assessed using either a DICOM reference reconstructed from treatment planning CT images or an SGRT reference acquired by the SGRT system. The systems differ in surface acquisition technology, camera configuration and the algorithm used to derive the correction (Table 1). The rigid/non‐rigid classification follows the vendor documentation and is a simplification—in Catalyst the displayed color map represents surface‐to‐surface distance whereas the computed correction may include non‐rigid terms—and because the phantom is rigid, results should not be attributed to the algorithm class. The overall study workflow is illustrated in Figure 1(b). (i) Upon receipt of the postal audit phantom, each institution assembled the phantom by attaching the surface component to a cubic phantom and performed a markerless treatment‐planning CT scan under their routine clinical imaging conditions. (ii) Using CT images in which the simulated tumor and gold markers were identifiable, a treatment plan with two orthogonal static fields (gantry angles 180° and 90°, photon energy ≥ 6 MV, field size 5 × 5 cm^2^, 500 MU per field) was generated and transferred to the treatment delivery system. (iii) Irradiation was performed using the following two SGRT‐based setup methods and the setup time required for each method was recorded by the institution.

- DICOM reference setup

First, the markerless setup was evaluated using a DICOM reference surface generated from the treatment planning CT images. After completing the setup, positional adjustments were made until the SGRT system displayed values as close as possible to 0 mm/0°. Irradiation was delivered with two orthogonal fields without additional image‐guided radiation therapy (IGRT).

B. SGRT reference setup

Subsequently, the markerless setup was evaluated using an SGRT reference surface acquired using the SGRT system in the treatment room. A cube phantom different from that used in step A was used. Immediately after IGRT‐based alignment of the embedded gold markers, the reference surface was captured and registered as the SGRT reference, and the phantom was then removed from the couch and repositioned using it. As in **step A**, positional adjustments were made until the SGRT system displayed values as close as possible to 0 mm/0°, followed by delivery of two orthogonal fields without additional IGRT.

**TABLE 1:** Specifications of the five SGRT systems as stated in the vendor documentation.

System	Surface acquisition principle	Camera configuration	Registration	Nominal accuracy
AlignRT	Structured light with stereo‐vision	3 cameras	Rigid	0.5 mm / 0.5°
Catalyst	Structured light (projected pattern)	3 or 1 camera	Non‐rigid	0.5–1 mm / 0.5°
ExacTrac Dynamic	Structured light with thermal imaging	1 camera	Rigid	Not specified
IDENTIFY	Structured light	3 cameras	Rigid	0.5–1 mm / 0.5°
VOXELAN	Light‐section method	1 camera	Rigid	1 mm / 1°

Finally, the research office compared the data obtained in **steps A** and **B**. After completing all procedures, the responsible staff at each participating institution completed a post‐study questionnaire via Google Forms. The questionnaire included items on years of SGRT experience, setup time for the current experiment (defined as the time from initiation of SGRT‐based position verification at the phantom setup position to acquisition of post‐alignment capture), region of interest (ROI) settings, surface recognition, IGRT technique, and radiotherapy treatment planning system (RTPS) used for surface generation. Each institution returned the phantom with a disk containing the planning CT images, the DICOM RT Plan, RT Dose and RT Structure data, and the captured images. No ROI size or shape was prescribed (Figure 2); ROI area, QA tolerances for isocenter coincidence, the residual IGRT error at capture and body‐contour thresholds were not recorded.

**FIGURE 2:**
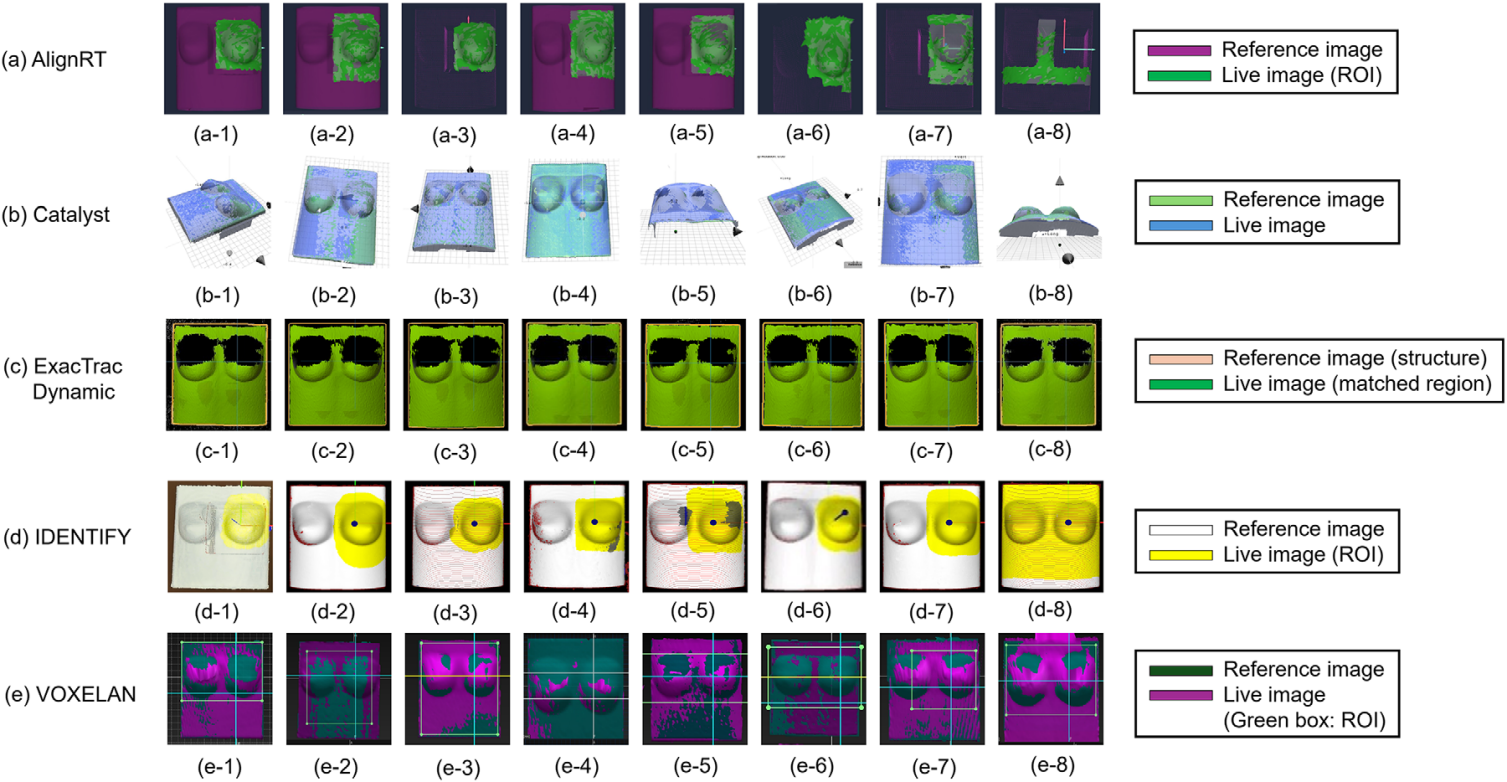
DICOM reference setup conditions submitted by each institution. From top to bottom, images from institutions using (a) AlignRT, (b) Catalyst, (c) ExacTrac Dynamic, (d) IDENTIFY, and (e) VOXELAN are shown.

## 3 STATISTICAL ANALYSIS

For each SGRT system, six‐degree‐of‐freedom (6DOF) static irradiation accuracy was evaluated separately for the DICOM and SGRT references. The three‐dimensional translational vector error, hereafter referred to as the 3D vector error, was defined as the primary evaluation metric. It was computed as the Euclidean norm √(ΔLR^2^ + ΔAP^2^ + ΔSI^2^) of the film‐derived residuals relative to the intended center; positive values denote left, anterior, cranial and clockwise. Signed axis‐specific values are also tested against zero with the one‐sample Wilcoxon signed‐rank test. The institution that remained out of tolerance and was not remeasured was excluded, leaving 39 DICOM and 31 SGRT setups. The 3D vector error and setup time were compared between the DICOM and SGRT references using the Wilcoxon signed‐rank test, given the relatively small sample size in each device group (*n* = 7–8) and the non‐normal distribution of the data, as assessed using the Shapiro–Wilk test.

Associations between multiple binary factors and either the 3D vector error or setup time were evaluated using multivariable linear regression, fitted to the 39 DICOM reference setups with the five binary factors entered simultaneously. These analyses were limited to the DICOM reference setup, as the DICOM reference was available for all systems. Each outcome was treated as a continuous dependent variable. Regression coefficients (β), representing adjusted mean differences, were estimated with 95% confidence intervals and *p*‐values. All statistical analyses were performed using MATLAB R2022b.

## 4 DECLARATION OF GENERATIVE AI AND AI‐ASSISTED TECHNOLOGIES

During the preparation of this manuscript, the authors used ChatGPT (GPT‐5.5 by OpenAI) and Claude (Opus 4.7) to enhance the readability and proofread the text. After using this service, the authors reviewed and edited the content as needed and took full responsibility for the content of the publication.

## 5 RESULTS

Table 2 summarizes the institutional background characteristics. Among the 40 participating institutions, 28 (70.0%) had at least 1 year of SGRT experience, and 11 (27.5%) had implemented markerless clinical practice. Three‐camera and single‐camera systems were used in 23 (57.5%) and 17 (42.5%) institutions, respectively. Most institutions used C‐arm linacs (37/40, 92.5%), and the treatment couch was connected to the SGRT system in 23 institutions (57.5%). A left breast‐only ROI was used in 17 institutions (42.5%), whereas both breasts or whole‐phantom ROIs were used in 23 (57.5%). For SGRT reference acquisition, kV‐CBCT with 6DOF alignment was the most common IGRT technique (18/32, 56.3%). Surface contours for DICOM reference generation were created mainly using Eclipse (25/40, 62.5%), followed by RayStation, Monaco, and Precision. The planning CT datasets submitted by the institutions had an in‐plane voxel size of 0.881–1.367 mm (median, 0.977 mm) and a slice thickness of 1.0–3.0 mm (median, 2.0 mm), and were acquired on scanners from three vendors (Table 2). The reconstruction field of view was 451–700 mm (median, 500 mm) and a tube voltage of 120 kV was used at 37 of 40 institutions.

**TABLE 2:** Institutional background characteristics (*n* = 40).

	Characteristic	*n* (%)
1. Clinical practice of SGRT		
	*SGRT experience*	
	≥ 1 year	28 (70)
	< 1 year	12 (30)
	*Implementation of markerless*	
	Markerless	11 (27.5)
	Marker‐based	29 (72.5)
2. Experimental conditions in this audit		
	*Camera condition*	
	3‐camera	23 (57.5)
	1‐camera	17 (42.5)
	*Treatment machine type*	
	C‐arm linac	37 (92.5)
	Ring‐type linac	3 (7.5)
	*Connection to treatment couch*	
	Connected	23 (57.5)
	Not connected	17 (42.5)
	*ROI setting*	
	Left breast only	17 (42.5)
	Both breasts / whole phantom	23 (57.5)
	*IGRT technique (for SGRT reference acquisition) (n = 32)*	
	kV‐CBCT (6DOF alignment)	18 (56.3)
	kV‐CBCT (3DOF alignment)	6 (18.8)
	kV 2D image (6DOF alignment)	4 (12.5)
	kV 2D image (3DOF alignment)	2 (6.3)
	MVCT	2 (6.3)
	*RTPS for surface contour generation*	
	Eclipse	25 (62.5)
	RayStation	9 (22.5)
	Monaco	4 (10.0)
	Precision	2 (5.0)
3. Planning CT parameters		
	In‐plane voxel size (median, 0.977 mm)	
	≤ 1.0 mm	26 (65.0)
	> 1.0 mm	14 (35.0)
	Slice thickness (median, 2.0 mm)	
	1.0–2.0 mm	29 (72.5)
	2.5–3.0 mm	11 (27.5)
	CT scanner vendor	
	Canon / Toshiba	21 (52.5)
	Siemens	15 (37.5)
	GE	4 (10.0)

A total of 40 institutions participated in the audit. The DICOM reference setup was performed at all 40 institutions, whereas the SGRT reference setup was performed at 32 institutions. At the initial audit, critical setup errors exceeding 5 mm occurred in four institutions, corresponding to an institution‐level error rate of 10.0%. For the DICOM reference setup, three institutions exceeded the predefined 5 mm action level. The confirmed causes were misunderstanding of the study protocol at one institution; incorrect assembly of the cube phantom at one institution; and insufficient commissioning between the CT scanner and the SGRT system at one institution. Repeat measurements were performed at the first two institutions, both of which subsequently achieved acceptable accuracy. The remaining institution was not remeasured, remained out of tolerance, and was excluded as an outlier from the quantitative analyses. For the SGRT reference setup, one institution exceeded the action level because of incorrect registration of the reference surface. A repeat measurement was performed at this institution, and acceptable accuracy was subsequently achieved.

Figure 2 shows the DICOM reference setup conditions submitted by each institution. Although variability in the ROI settings was observed across institutions, the phantom was successfully recognized at all institutions.

Table 3 summarizes the comparison of 3D vector error and setup time for each system. The DICOM reference tended to show larger errors than the SGRT reference across some devices. This difference was statistically significant for all devices combined, excluding ExacTrac Dynamic, with mean ± SD values of 1.54 ± 0.91 mm and 1.20 ± 0.61 mm, respectively (*p* = 0.043). Histograms of the 6DOF distributions for all devices and each individual system are shown in **Figure** **3**. Rotations were small: only 1 of 117 (0.9%) rotational measurements with the DICOM reference and 2 of 93 (2.2%) with the SGRT reference exceeded ± 1°, no signed component differed between the references (all p ≥ 0.10), and none differed between the five systems (Kruskal–Wallis p ≥ 0.21). Axis‐specific signed results are summarized in **Table** **4**. With the DICOM reference, 87.2%, 82.1%, and 61.5% of the LR, AP, and SI residuals were within ± 1 mm; the corresponding proportions with the SGRT reference were 80.6%, 96.8%, and 67.7%. In the 31 institutions with both references, the LR residuals were centered at zero (DICOM, −0.02 ± 0.70 mm, *p* = 0.992; SGRT, −0.03 ± 0.83 mm, *p* = 0.977). A systematic offset in the caudal direction was present in the SI residuals with both references (DICOM, −0.50 ± 1.29 mm, *p* = 0.008; SGRT, −0.37 ± 0.87 mm, *p* = 0.009), and the two references did not differ in this component (*p* = 0.854). The AP residuals were offset anteriorly with the DICOM reference (0.28 ± 0.88 mm, *p* = 0.048) but not with the SGRT reference (0.10 ± 0.51 mm, *p* = 0.264). The difference between the two references was carried almost entirely by the AP component: the mean absolute AP error was 0.36 mm larger with the DICOM reference (*p* = 0.002), against 0.19 mm for SI (*p* = 0.664), −0.06 mm for LR (*p* = 0.706) and 0.34 mm for the 3D vector error.

**TABLE 3:** Comparison of 3D vector error and setup time between the DICOM reference and the SGRT reference for each SGRT system. Values are mean ± SD. *P*‐values were calculated using the Wilcoxon signed‐rank test for paired comparisons between the two references. *p < 0.05.

System	3D vector error (mm) DICOM reference	3D vector error (mm) SGRT reference	*p*	Setup time (s) DICOM reference	Setup time (s) SGRT reference	*p*
AlignRT (*n* = 8)	1.16 ± 0.35	0.82 ± 0.43	0.055	50.38 ± 19.46	52.12 ± 23.24	0.570
Catalyst (*n* = 8)	1.51 ± 0.59	1.35 ± 0.36	0.641	109.38 ± 55.85	93.88 ± 50.75	0.469
ExacTrac Dynamic (*n* = 8)	1.79 ± 0.68	—	—	63.00 ± 36.76	—	—
IDENTIFY (*n* = 8)	1.11 ± 0.59	1.21 ± 0.50	0.844	84.50 ± 25.56	86.00 ± 23.84	0.266
VOXELAN (*n* = 7)	2.49 ± 1.31	1.45 ± 0.94	0.078	102.57 ± 43.63	96.29 ± 30.70	0.688
All devices except ExacTrac Dynamic (*n* = 31)	1.54 ± 0.91	1.20 ± 0.61	0.043*	86.19 ± 43.57	81.61 ± 37.09	0.804

**FIGURE 3:**
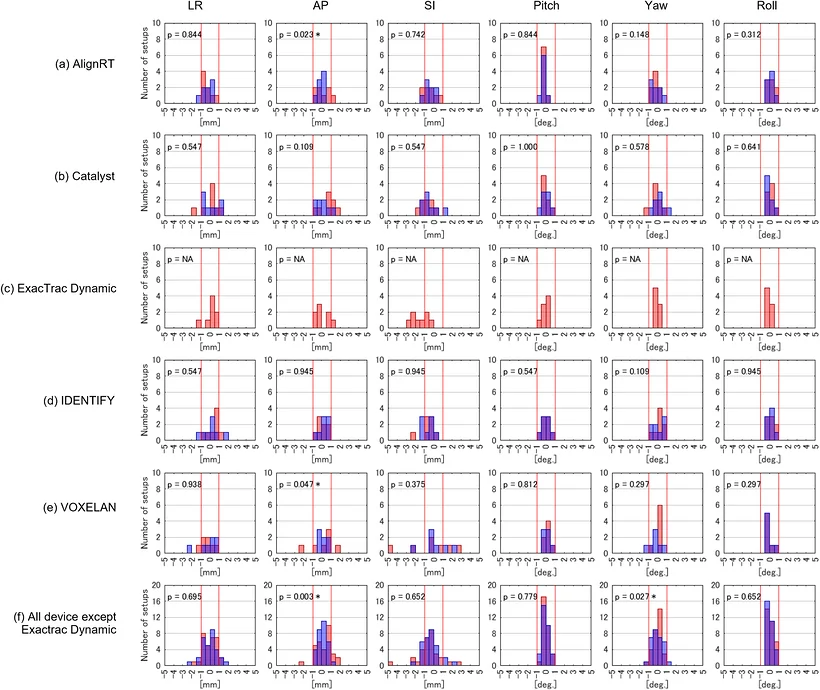
Histograms of six‐degree‐of‐freedom irradiation positional accuracy. Rows are the SGRT systems—(a) AlignRT, (b) Catalyst, (c) ExacTrac Dynamic, (d) IDENTIFY, (e) VOXELAN and (f) all devices except ExacTrac Dynamic—and columns are the translational (LR, AP, SI; mm) and rotational (pitch, yaw, roll; degrees) components. Red bars, DICOM reference; blue bars, SGRT reference. Vertical red lines, ± 1 mm and ± 1°. Bin width, 0.50 mm and 0.50°. Each panel gives the p value of the Wilcoxon signed‐rank test between the two references (*p < 0.05); NA indicates that the SGRT reference was not available.

**TABLE 4:** Axis‐specific accuracy for the DICOM (*n* = 39) and SGRT (*n* = 31) references, which cover different institutions. Values are mean ± SD and median (interquartile range, IQR); positive values denote left, anterior, cranial and clockwise. Within tolerance, ± 1 mm for translations and ± 1° for rotations; not applicable to the 3D vector.

Axis	DICOM (*n* = 39): mean ± SD	DICOM: median (IQR)	DICOM: within tolerance, n (%)	SGRT (*n* = 31): mean ± SD	SGRT: median (IQR)	SGRT: within tolerance, n (%)
LR (mm)	0.00 ± 0.66	0.18 (−0.51 to 0.54)	34/39 (87.2)	−0.03 ± 0.83	0.02 (−0.58 to 0.47)	25/31 (80.6)
AP (mm)	0.24 ± 0.84	0.34 (−0.36 to 0.93)	32/39 (82.1)	0.10 ± 0.51	0.10 (−0.15 to 0.47)	30/31 (96.8)
SI (mm)	−0.71 ± 1.27	−0.67 (−1.22 to −0.10)	24/39 (61.5)	−0.37 ± 0.87	−0.46 (−0.96 to 0.01)	21/31 (67.7)
Pitch (°)	−0.06 ± 0.34	−0.05 (−0.27 to 0.13)	39/39 (100.0)	−0.05 ± 0.40	−0.07 (−0.33 to 0.22)	31/31 (100.0)
Yaw (°)	−0.04 ± 0.41	0.01 (−0.33 to 0.23)	38/39 (97.4)	−0.01 ± 0.58	−0.08 (−0.50 to 0.45)	29/31 (93.5)
Roll (°)	0.07 ± 0.34	0.00 (−0.17 to 0.33)	39/39 (100.0)	0.09 ± 0.32	−0.01 (−0.14 to 0.33)	31/31 (100.0)
3D vector (mm)	1.59 ± 0.87	1.30 (1.00 to 2.01)	—	1.20 ± 0.61	1.25 (0.73 to 1.48)	—

Setup time is also summarized in Table 3. No statistically significant differences were observed for any device. For all devices combined, excluding ExacTrac Dynamic, the mean ± SD setup times were 86.19 ± 43.57 s and 81.61 ± 37.09 s for the DICOM and SGRT references, respectively (*p* = 0.804).

Table 5 summarizes the multivariable linear regression analyses of factors associated with 3D vector error and setup time. For 3D vector error, camera condition was the only significant factor; the 1‐camera condition was associated with a larger error than the 3‐camera condition (β = 0.90 mm; 95% CI, 0.17–1.63 mm; *p* = 0.017). No other factors were significantly associated with 3D vector error. For setup time, none of the evaluated factors showed a statistically significant association.

**TABLE 5:** Multivariable linear regression analyses of factors associated with 3D vector error and setup time, fitted to the 39 DICOM reference setups with the five factors entered simultaneously. β denotes the adjusted mean difference of the comparison category relative to the reference category. *p < 0.05.

Factor	Reference category	Comparison category	3D vector error β (mm)	95% CI (mm)	p	Setup time β (s)	95% CI (s)	p
SGRT experience	< 1 year	≥ 1 year	0.09	−0.64 to 0.82	0.799	−23.53	−63.93 to 16.87	0.245
Implementation of markerless	Marker‐based	Markerless	0.30	−0.41 to 1.01	0.395	−16.97	−56.18 to 22.24	0.385
Camera condition	3‐camera	1‐camera	0.90	0.17 to 1.63	0.017*	8.44	−31.82 to 48.70	0.672
Device connection	Not connected	Connected	−0.26	−0.81 to 0.30	0.360	−5.86	−36.75 to 25.02	0.702
ROI setting	Both breasts	Left breast	−0.09	−0.69 to 0.52	0.775	3.25	−30.31 to 36.80	0.845

Furthermore, Figure 4 shows a scatter plot between 3D vector error and setup time. No clear correlation was observed between the two (Spearman ρ = 0.16, *p* = 0.324 for the DICOM reference and ρ = 0.24, *p* = 0.202 for the SGRT reference).

**FIGURE 4:**
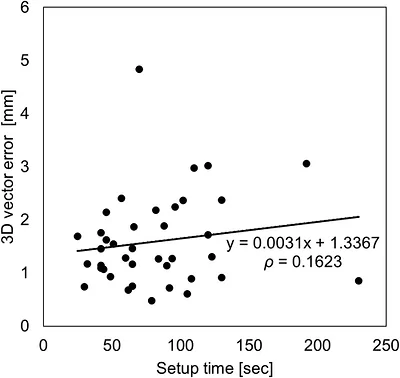
Scatter plot showing the relationship between 3D vector error [mm] and setup time [s]. Results are shown for the DICOM reference. The fitted regression line and Spearman's rank correlation coefficient (ρ) are shown.

## 6 DISCUSSION

To the best of our knowledge, this study represents the first large‐scale multicenter postal audit of skin markerless SGRT. Our results indicate that skin markerless SGRT may carry an approximately 10% risk of critical positional errors exceeding 5 mm in the 3D vector. These findings provide important information for risk management in clinical implementation. In addition, positional errors were larger with the DICOM reference than with the SGRT reference in the combined analysis excluding ExacTrac Dynamic. The two references are not generated by symmetric processes, however: the DICOM reference propagates CT acquisition, body‐contour thresholding, mesh export and the CT‐to‐linac transfer, whereas the SGRT reference bypasses these but embeds the accuracy of the IGRT chain (Table 6). The smaller error with the SGRT reference therefore reflects workflow properties rather than superiority of surface registration, and neither reference is an error‐free ground truth. One contributor is that DICOM reference surfaces are derived from CT images, so that the accuracy of body contour delineation depends on the RTPS and its threshold settings,^19^ a trend that has also been reported in previous studies on the VOXELAN system.^18^ The axis‐specific analysis localizes this effect: the difference between the references was carried almost entirely by the anterior–posterior component, for which the mean absolute error was 0.36 mm larger with the DICOM reference (*p* = 0.002) against 0.34 mm in the 3D vector error, while left–right and superior–inferior contributed little (−0.06 mm, *p* = 0.706, and 0.19 mm, *p* = 0.664). This is the direction in which a body‐contour threshold displaces the reconstructed surface, and the depth direction least well constrained for a ceiling‐mounted camera. Supporting this, the reference‐related component differed between planning systems (Eclipse, 0.06 mm; Monaco, −0.22 mm; RayStation, 0.97 mm; Precision, 1.73 mm; *p* = 0.016) and the anterior–posterior error was associated with in‐plane voxel size (ρ = 0.33, *p* = 0.041) but not slice thickness (ρ = −0.19, *p* = 0.306). These post hoc analyses are hypothesis‐generating. In the inter‐device comparisons, the Catalyst and VOXELAN systems tended to exhibit larger errors, as shown in Table 3 and Figure 3. Because both setups used the same device, the within‐device difference isolates the DICOM‐reference component while the SGRT‐reference level reflects device‐related factors. For Catalyst the error was elevated with both references and the within‐device difference was small (1.51 vs. 1.35 mm; Δ = 0.16 mm), indicating a device‐ or algorithm‐related contribution; for VOXELAN the SGRT‐reference error was only modestly elevated (1.45 mm) while the DICOM‐reference error was much larger (2.49 mm; Δ = 1.04 mm), the largest reference‐related component observed. A previous comparative study also reported inter‐device differences.^20^ However, camera resolution and the registration algorithm are device properties that should affect both references equally and therefore cannot by themselves explain why the errors were substantially larger with the DICOM reference; the manner in which the body structure is imported, decimated, smoothed, and re‐triangulated when a DICOM reference is loaded differs between vendors and is a more plausible dominant contributor for this system. Because these comparisons are post hoc and based on small paired samples (*n* = 7–8 per device), they should be regarded as hypothesis‐generating. In particular, VOXELAN has been reported to show increased errors in the anterior–posterior direction, likely due to interpolation‐based surface estimation.^20^ However, although the DICOM reference tended to show larger errors overall, the SGRT reference can also introduce critical systematic positional errors relative to the planned treatment position if it is acquired incorrectly. In fact, this occurred at one of the 32 institutions that performed the SGRT reference setup (3.1%).

**TABLE 6:** Error sources that differ between the two reference surface workflows. “Yes” indicates that the uncertainty is carried by that workflow; sources common to both are omitted.

Error source	DICOM reference	SGRT reference
1. CT acquisition and reconstruction (voxel size, slice thickness, kernel)	Yes	No
2. Body‐contour definition and threshold setting in the treatment planning system	Yes	No
3. Mesh generation and export, and re‐import by the SGRT system	Yes	No
4. CT‐to‐linac coordinate transfer	Yes	No
5. Imaging‐to‐treatment isocenter coincidence and couch correction	No	Yes
6. Residual error of the IGRT alignment at which the reference was captured	No	Yes

Because the SGRT reference is captured immediately after IGRT‐based alignment, any residual error in that chain is written into the reference as a systematic offset that recurs at every setup and cannot be detected by the system, particularly under three‐degree‐of‐freedom (3DOF) alignment (Table 2). A caudal offset of similar magnitude was nevertheless present in the superior–inferior component with both references (−0.50 ± 1.29 and −0.37 ± 0.87 mm; *p* = 0.008 and 0.009; between‐reference *p* = 0.854). Since the SGRT reference bypasses CT‐based surface generation, an offset of this size in both workflows more plausibly arises from a step they share—the common phantom and the film‐based determination of the radiation center, a residual isocenter offset or couch sag, or alignment to the room lasers. We therefore report it as a residual uncertainty of the audit chain rather than a property of either workflow; it limits the absolute accuracy but not the paired comparison.

In this study, the ceiling‐mounted 1‐camera condition was significantly associated with a larger 3D vector error than the ceiling‐mounted 3‐camera condition. This finding is consistent with the basic principles of optical surface reconstruction. Structured‐light systems estimate 3D surface geometry using optical triangulation,^21^ and accurate depth estimation requires reliable correspondence between image points and projected or viewed patterns.^22^ Therefore, a single‐camera view may be more susceptible to limited field of view and self‐occlusion, whereas multi‐view reconstruction can integrate information from multiple calibrated images and compensate for sparse or missing surface data.^23^ These principles support the interpretation that a 3‐camera configuration may provide more robust surface registration in markerless SGRT.

This is consistent with AAPM TG‐302 and the ESTRO‐ACROP guideline, which indicate that SGRT accuracy depends on multiple factors, including camera field of view, surface recognition, workflow, commissioning, and QA.^5^, ^6^ In contrast, Kügele et al. reported no significant difference in setup results between single‐camera and three‐camera systems in breast irradiation.^24^ The discrepancy between their findings and ours may be attributable to the multicenter and multivendor markerless audit design of the present study, as well as our evaluation of static irradiation accuracy using a breast‐shaped phantom rather than routine clinical setup results.

Because SGRT registers the external surface, a residual rotation θ displaces a target a distance d away by about d·tan θ; with *d* = 139 mm, 1° corresponds to 2.4 mm and the median residual rotation here (0.56°) to 1.4 mm. Testing this directly gave no evidence that it drives the observed errors (ρ = −0.33, *p* = 0.039 for DICOM; ρ = 0.05, *p* = 0.779 for SGRT). Lever‐arm propagation is geometrically possible but not dominant here, and the geometry is conservative relative to a shallower clinical breast isocenter, so the absolute values should not be transferred to breast irradiation.

In contrast, none of the evaluated factors showed a significant association with setup time. This may be because setup time is influenced by multiple practical factors, including not only camera configuration but also institutional workflow, operator judgment, and verification procedures. Furthermore, the absence of a clear association between setup time and 3D vector error suggests that spending more time on setup does not necessarily improve setup accuracy and that technical or system‐specific limitations may exist. Therefore, in clinical SGRT practice, each institution should establish an appropriate balance between setup accuracy and workflow efficiency.

Five practical recommendations follow. First, verify the DICOM reference before clinical markerless use: the body‐contour threshold and the exported mesh should be checked against the planning CT for each planning‐system and SGRT‐system combination at commissioning, and after any change to the planning system or contouring protocol. Second, prefer multi‐camera configurations, since a single camera was independently associated with a 0.90 mm larger error. Third, do not use setup time as a surrogate for accuracy. Fourth, treat the SGRT reference as safety‐critical: an incorrectly acquired reference produces an offset reproduced at every fraction that the system cannot detect, so acquisition should follow a written procedure with an independent check. Fifth, maintain an end‐to‐end action level and periodic external audit, since every critical error here was an implementation error undetectable by component‐level QA.

This study has several limitations. First, due to the end‐to‐end nature of the evaluation, the results depend not only on the registration accuracy of the SGRT systems, but also on the accuracy of the treatment delivery systems. Second, although the ExacTrac Dynamic has since been updated to allow the acquisition of the SGRT reference, this capability was not available at the time the study was initiated and, therefore, could not be evaluated. Third, no baseline cross‐check between Cube A and Cube B was performed, so a cube‐specific offset cannot be excluded, although it would be common to all institutions and would not explain the axis‐specific pattern in **Table** **4**. Fourth, the reproducibility of attaching the breast‐shaped component was not quantified, yet incorrect assembly caused one out‐of‐tolerance result. Fifth, the film‐based uncertainty rests on three repetitions. Sixth, setup time was self‐reported, with one measurement per setup. Seventh, the body‐contour thresholds, the QA tolerance for isocenter coincidence, the residual IGRT error at reference acquisition, the SGRT software version and the ROI area were not collected. Eighth, no ROI size or shape was prescribed, which captured real‐world variability but limits between‐institution comparison. Ninth, a single, relatively deep isocenter was used; repeating the audit at several depths would separate intrinsic registration performance from propagation to depth. Future studies should extend the evaluation to dynamic treatment scenarios, including latency, respiratory‐gated delivery, and intrafraction motion management.

## 7 CONCLUSIONS

This postal audit showed that markerless SGRT can generally achieve millimeter‐level static irradiation accuracy across different commercial systems when appropriately implemented, although superior–inferior residuals were often outside 1 mm. However, critical setup errors exceeding 5 mm occurred in 10.0% of institutions at the initial audit, indicating that markerless SGRT is not free from clinically relevant systematic errors. The DICOM reference tended to show larger 3D vector errors than the SGRT reference, suggesting that the choice of reference surface workflow, rather than the registration algorithm itself, can influence setup accuracy. This difference was confined to the anterior–posterior component, identifying CT‐based surface generation as a principal target for commissioning, whereas a superior–inferior offset common to both workflows indicates a residual uncertainty in the audit chain itself. The 1‐camera condition was independently associated with larger 3D vector errors, highlighting the importance of camera configuration. Setup time was associated with none of the evaluated factors and was not a surrogate for accuracy. Commissioning, standardized reference surface acquisition, routine QA and independent third‐party audits are therefore essential for safe clinical implementation.

## AUTHOR CONTRIBUTION

**Masahide Saito**: Conceptualization, methodology, validation, formal analysis, investigation, data curation, writing—original draft, supervision, project administration. **Ryohei Yamauchi**: Conceptualization, methodology, validation, formal analysis, investigation, data curation, writing—review and editing. **Hironori Kojima**: Methodology, investigation, writing—review and editing, supervision. **Yoshinobu Shimohigashi**: Methodology, investigation, writing—review and editing. **Takeshi Ohno**: Methodology, investigation, writing—review and editing. **Yoshifumi Oku**: Methodology, investigation, writing—review and editing. **Shinobu Kumagai**: Methodology, investigation, writing– review and editing. **Naoki Tohyama**: Methodology, investigation, writing—review and editing. **Masahiko Kurooka**: Methodology, investigation, writing—review and editing. **Eriko Saito**: Methodology, investigation, writing—review and editing. **Masataka Sakamoto**: Methodology, investigation, writing—review and editing. **Yusuke Ueshima**: Methodology, investigation, writing—review and editing. **Takayuki Kanai**: Methodology, investigation, writing—review and editing. **Naoki Hayashi**: Methodology, investigation, writing—review and editing. **Tatsunori Saito**: Methodology, investigation, writing –review and editing. **Hiroki Katayama**: Methodology, investigation, writing—review and editing. **Makoto Sasaki**: Methodology, investigation, writing—review and editing. **Tomoki Kitagawa**: Methodology, investigation, writing—review and editing. **Hidekazu Suzuki**: Methodology, investigation, writing—review and editing. **Chie Kurokawa**: Methodology, investigation, writing—review and editing, supervision. **Hiroshi Onishi**: Methodology, writing—review and editing, supervision, project administration.

## ETHICAL APPROVAL

The participating institutions were recruited through a nationwide survey questionnaire approved by the Institutional Review Board of St. Luke's International Hospital (approval No. No. 24‐RC041) and informed consent for participation was obtained.

## CONFLICT OF INTEREST STATEMENT

M. Saito and H. Onishi have received an SGRT system (VOXELAN) on loan from Hamano engineering and ERD as part of a collaborative research agreement. M. Sasaki is affiliated with an institution that serves as a reference site for VisionRT, the manufacturer of the SGRT system (AlignRT). No personal financial compensation has been received.

## Data Availability

Research data are stored in an institutional repository and will be shared upon request to the corresponding author.
